# Chronic Hypercapnic Respiratory Failure in COPD in Lithuania: National Registry Trends and Prehospital Emergency-Care Professionals’ Knowledge of Hypercapnia and Oxygen Therapy

**DOI:** 10.3390/diagnostics16172782

**Published:** 2026-08-29

**Authors:** Rasa Gauronskaitė, Anrenas Karvelis, Edvardas Danila, Rolandas Zablockis

**Affiliations:** 1 Clinic of Chest Diseases, Immunology and Allergology, Faculty of Medicine, Institute of Clinical Medicine, Vilnius University, LT-01513 Vilnius, Lithuania; 2 Department of Health Sciences and Technologies, St. Ignatius Loyola College, LT-44286 Kaunas, Lithuania

**Keywords:** chronic obstructive pulmonary disease, hypercapnia, hypercapnic respiratory failure, oxygen therapy, emergency medical services, prehospital emergency care, health knowledge, electronic health records, Lithuania

## Abstract

**Background/Objectives**: Hypercapnic respiratory failure (HRF) is a serious complication of chronic obstructive pulmonary disease (COPD) and is associated with increased morbidity, mortality, and healthcare utilisation. National HRF trends among patients with COPD remain poorly characterised in Lithuania, and the knowledge of prehospital emergency-care professionals regarding oxygen therapy and hypercapnia has not been evaluated. **Methods**: This study comprised two components. First, a cross-sectional online survey assessed knowledge of COPD, hypercapnia, and oxygen therapy among prehospital emergency-care professionals (EMS physicians, emergency nurses, EMS paramedics, and paramedics); three questionnaire items whose designated correct answers could not be unambiguously supported were excluded from the knowledge score, and the original 10-item score was retained as a sensitivity analysis. Second, nationwide electronic health record data from the Lithuanian national health record system (ESPBI IS, 2020–2023) were analysed retrospectively: patients with COPD and concomitant chronic HRF were identified using ICD-10-AM diagnostic codes, and the number of patients with a recorded J96.11 diagnosis irrespective of underlying disease was obtained as a national denominator. Proportions are reported with 95% confidence intervals and temporal trends as absolute changes in percentage points; multivariable logistic regression was used to estimate odds ratios adjusted for age group and sex. Between-year differences were tested using the χ^2^ test with Holm-adjusted pairwise comparisons, and the association between sex and chronic HRF was expressed as an odds ratio. All tests were two-sided, and *p* < 0.05 was considered significant. **Results**: Among the 65 prehospital emergency-care professionals surveyed, knowledge of hypercapnia was relatively high: 78.5% (95% CI 67.0–86.7) correctly defined it and 72.3% (95% CI 60.4–81.7) identified its symptoms, whereas 58.5% (95% CI 46.3–69.6) identified the recommended SpO_2_ target range of 88–92%. Respondents answered a median of 5 of the 7 scored knowledge items correctly (IQR 4–6). Total knowledge scores differed by professional role (*p* = 0.034) but not by years of experience, sex, or frequency of COPD contact; the sensitivity analysis using the original 10-item score gave the same pattern (*p* = 0.006). In the national electronic health record data, the COPD population increased from 27,239 to 32,446 (+19.1%) between 2020 and 2023, while the number of COPD patients with a recorded chronic HRF diagnosis rose from 160 to 291 (+81.9%). The proportion rose from 0.59% (95% CI 0.50–0.69) to 0.90% (95% CI 0.80–1.01), an absolute increase of 0.31 percentage points (95% CI 0.17–0.45; risk ratio 1.53, 95% CI 1.26–1.85). Men had higher odds than women after adjustment for age (adjusted OR 1.55, 95% CI 1.28–1.88), and adjusted odds were lowest in patients aged 80 years or older. Across the four annual cross-sections, COPD accounted for 41.5% of patient-year observations with a recorded J96.11 diagnosis nationally. **Conclusions**: Knowledge of COPD, hypercapnia, and oxygen therapy varied among the prehospital emergency-care professionals surveyed, with one of the lowest proportions of correct responses observed for the recommended target oxygen saturation range. National electronic health record data showed an increase in the proportion of patients with COPD who had a recorded diagnosis of chronic HRF between 2020 and 2023. These components were analysed independently, and no relationship between professional knowledge and temporal trends in recorded diagnoses was assessed. The electronic health record findings support continued monitoring and recognition of chronic HRF in COPD, whereas the survey findings support further evaluation of targeted education on oxygen therapy in the prehospital setting.

## 1. Introduction

Chronic obstructive pulmonary disease (COPD) is a major cause of morbidity and mortality worldwide, and imposes a substantial burden on healthcare systems. The global prevalence of COPD is projected to continue to rise over the coming decades due to ageing populations and ongoing exposure to risk factors [1]. Patients with advanced COPD may account for a substantial share of healthcare utilisation, in part due to recurrent exacerbations and respiratory complications [2]. Hypercapnic respiratory failure (HRF) is an important complication of advanced COPD. Approximately one-quarter of patients with severe COPD may have chronic hypercapnia, many of whom remain unrecognised and untreated [3]. Chronic hypercapnia develops as a result of progressive ventilatory impairment, dynamic hyperinflation, respiratory muscle dysfunction, and ventilation–perfusion mismatch [4]. Previous studies have shown that chronic hypercapnia is associated with more severe airflow limitation, increased exacerbation frequency, and worse clinical outcomes [4,5]. Oxygen therapy is a cornerstone of managing acute respiratory deterioration in patients with COPD. However, patients with COPD and chronic hypercapnia are particularly vulnerable to the adverse effects of excessive oxygen administration, which may precipitate or worsen hypercapnia through mechanisms such as worsening ventilation–perfusion mismatch and the Haldane effect [6,7]. Current guidelines therefore recommend titrated oxygen therapy to target an oxygen saturation of 88–92% in patients at risk of HRF [8,9]. Nevertheless, studies in both prehospital and hospital settings have shown frequent deviations from these recommendations and persistent challenges with oxygen titration in clinical practice [10,11]. Prehospital emergency-care professionals are often the first to assess patients with acute exacerbations of COPD and to initiate oxygen therapy. Given the clinical consequences of HRF and the growing burden of advanced COPD, the knowledge of prehospital emergency-care professionals responsible for the initial assessment and oxygen management of these patients is particularly important. Previous studies have identified knowledge gaps regarding emergency oxygen therapy among healthcare professionals [12,13,14,15]. However, these studies have largely been conducted in hospital settings or in mixed groups of clinicians, and knowledge specifically concerning the recognition of hypercapnia and the safe administration of oxygen among prehospital emergency-care professionals—particularly in relation to national trends in chronic HRF—has received little attention. In addition, data from Lithuania are lacking. Therefore, this study aimed to assess knowledge of COPD, hypercapnia, and oxygen therapy among prehospital emergency-care professionals in Lithuania and to describe national trends in chronic HRF among patients with COPD from 2020 to 2023.

## 2. Materials and Methods

### 2.1. Data Source

The survey was conducted in April–May 2025 using an anonymous online questionnaire. The questionnaire was distributed electronically via closed professional Facebook groups used by Lithuanian prehospital emergency care professionals. Participation was voluntary and anonymous, and no personal or identifying data were collected. Because the questionnaire was distributed through social media groups, the number of professionals who viewed the invitation could not be determined, and a formal response rate could not be calculated. In total, 65 professionals completed the questionnaire. The questionnaire was developed by the authors based on current clinical guidelines and previously published literature, and was not formally validated. The questionnaire consisted of demographic and experience questions followed by 10 single-answer knowledge questions covering knowledge of COPD, hypercapnia, oxygen therapy, and the acute management of COPD exacerbations, and one multiple-response question on COPD features (Appendix A). During revision, three single-answer items were reviewed against current guidance and excluded from the knowledge score because their designated correct answers could not be unambiguously supported. Item 7 (the effect of excessive oxygen administration) was excluded because its keyed answer reflected the hypoxic-drive concept, whereas oxygen-induced hypercapnia in COPD is now attributed principally to ventilation–perfusion mismatch and the Haldane effect [6,7], and because one distractor was also factually correct. Item 8 was excluded because increased peak expiratory flow is a physiological measurement rather than a symptom, which is inconsistent with the item stem. Item 11 was excluded because none of the response options corresponds to the guideline-recommended escalation for patients at risk of hypercapnic respiratory failure, in whom a 24–28% Venturi mask is recommended rather than a reservoir mask [9]. The knowledge score was therefore calculated from the remaining seven single-answer items. Responses to the three excluded items are reported in Appendix A but were not scored or interpreted. The epidemiological component used nationwide electronic health record data from the Electronic Health Services and Cooperation Infrastructure Information System (ESPBI IS; Lith. Elektroninė sveikatos paslaugų ir bendradarbiavimo infrastruktūros informacinė sistema), the Lithuanian national electronic health record system, for 2020–2023. Diagnosis records were drawn from two mandatory ESPBI IS document types—the E025 outpatient visit description and the E003 inpatient discharge summary (epicrisis). The data were requested and provided under the Lithuanian Law on Health Data Reuse through the State Data Agency, which retrieved, de-identified and supplied the records; analyses were performed within the State Health Data Platform, a secure processing environment based on the Palantir Foundry platform (Palantir Technologies Inc., Denver, CO, USA). Patients with COPD were identified on the basis of a recorded ICD-10-AM diagnosis of J44.0, J44.1, J44.8, or J44.9. Among these patients, chronic HRF was identified by the co-occurring ICD-10-AM code J96.11. Under ICD-10-AM (used nationally in Lithuania), code J96.11 denotes chronic type II (hypercapnic) respiratory failure. In addition, the annual number of patients with a recorded J96.11 diagnosis irrespective of underlying disease was obtained, providing a national denominator against which the COPD subgroup could be placed in context. Demographic variables for patients were limited to age and sex.

### 2.2. Study Population

This study comprised two populations. The survey population comprised prehospital emergency care professionals in Lithuania who voluntarily completed the anonymous questionnaire. This included physicians working in emergency medical service (EMS) teams, emergency nurses (SMPSS), EMS paramedics (SMPP), and paramedics. The epidemiological population comprised all patients with a recorded diagnosis of COPD in ESPBI IS during the 2020–2023 period. For contextual comparison, a second national group was also extracted: all patients with a recorded J96.11 diagnosis during the same period, irrespective of the underlying disease. This group was used to describe the share accounted for by COPD and to compare age and sex distributions between patient-year observations with and without a concurrent COPD diagnosis. All counts refer to unique patients within each calendar year, not to diagnosis records or treatment episodes; figures pooled across the four study years therefore represent patient-year observations.

### 2.3. Variables and Definitions

Survey variables included respondents’ demographic and professional characteristics (sex, professional role, and years of work experience) as well as knowledge of COPD, hypercapnia, oxygen therapy, and the management of COPD exacerbations (Appendix A). For the epidemiological component, COPD and chronic HRF were identified using the ICD-10-AM codes specified above. Annual analyses included the number of patients with a recorded COPD diagnosis, the number and proportion of these patients who also had a recorded chronic HRF diagnosis, and their distribution by age group and sex.

### 2.4. Outcomes

For the survey component, the primary outcome was the total knowledge score, calculated from the seven scored single-answer items remaining after the exclusions described in Section 2.1; the original 10-item score was retained as a sensitivity analysis. Secondary survey outcomes were the proportion of correct responses to each item and the distribution of scores across professional groups. For the epidemiological component, outcomes were the annual number of patients with a recorded COPD diagnosis and the number and proportion of these patients with a recorded chronic HRF diagnosis from 2020 to 2023; the absolute change in this proportion over the study period, overall and stratified by sex; the age and sex distribution of patients with chronic HRF; and the annual number of patients with a recorded J96.11 diagnosis irrespective of underlying disease, used as a national denominator.

### 2.5. Statistical Analysis

In the electronic health record component, categorical variables were summarised as frequencies and percentages. Because survey knowledge scores were not normally distributed, they were summarised as medians with interquartile ranges (IQR) and compared using non-parametric tests. For the survey, a total knowledge score (0–7) was calculated for each respondent as the number of correctly answered single-answer knowledge items remaining after the exclusions described in Section 2.1; the multiple-response item on COPD features was analysed separately at the item level, as the proportion of respondents selecting each feature. As a sensitivity analysis, all score-based comparisons were repeated using the original 10-item score to confirm that the exclusions did not alter the direction or significance of the findings. Differences in knowledge scores across professional roles and experience categories were assessed using the Kruskal–Wallis test, while differences by sex and by self-reported frequency of contact with COPD exacerbations were assessed using the Mann–Whitney U test. For the epidemiological component, between-year differences in the proportion of COPD patients with a recorded chronic HRF diagnosis were assessed using the χ^2^ test with Holm-adjusted pairwise post hoc comparisons. The association between sex and HRF was evaluated using odds ratios (ORs) with 95% confidence intervals (CIs). Proportions are reported with 95% confidence intervals calculated by the Wilson method. Between-year changes in the proportion of COPD patients with a recorded chronic HRF diagnosis are reported as absolute differences in percentage points with 95% confidence intervals, and as risk ratios with 95% confidence intervals. All statistical tests were two-sided, and *p* < 0.05 was considered statistically significant. Analyses were performed using IBM SPSS Statistics (version 31; IBM Corp., Armonk, NY, USA) and Python 3 (Python Software Foundation, Beaverton, OR, USA) for supplementary statistical computations. The multivariable logistic regression model was fitted with the presence of a recorded chronic HRF diagnosis among patients with COPD as the outcome and age group (<50, 50–59, 60–69, 70–79 and ≥80 years) and sex as explanatory variables, with the <50 years group and female sex as reference categories; results are reported as adjusted odds ratios with 95% confidence intervals. Age-group and sex distributions among patient-year observations with a recorded J96.11 diagnosis were compared according to concurrent COPD status using the χ^2^ test.

### 2.6. Use of Generative Artificial Intelligence

Generative artificial intelligence (Claude Opus 5, Anthropic, San Francisco, CA, USA) was used to assist with figure preparation, table formatting, and language editing. It was not used for study design, data collection, or statistical analysis. All AI-assisted outputs were reviewed and verified by the authors.

## 3. Results

### 3.1. Knowledge of COPD, Hypercapnia, and Oxygen Therapy

The characteristics of the 65 survey respondents—professional role, experience, sex, and contact frequency with COPD exacerbations by group—are summarised in Table 1.

**Figure 1:**
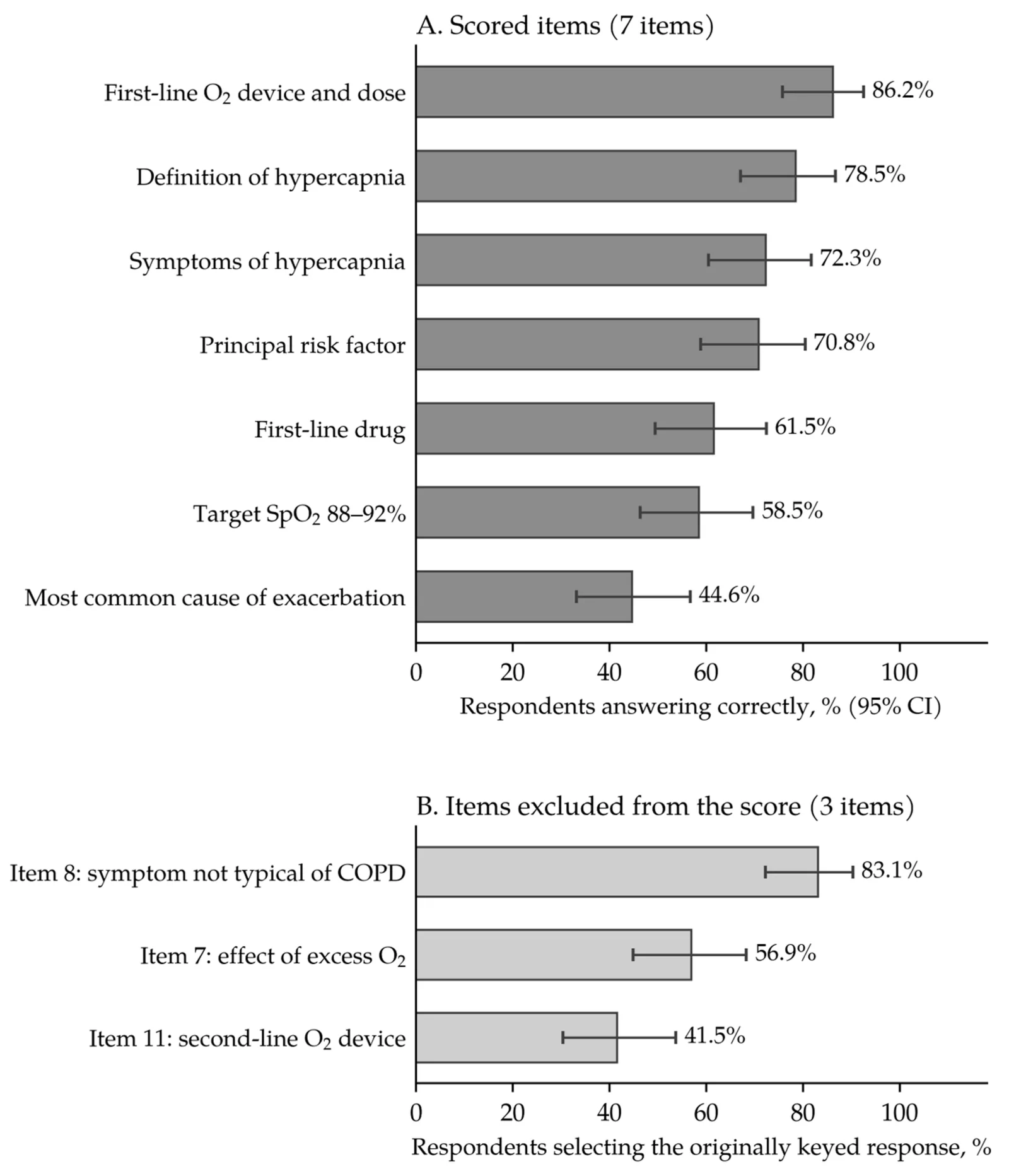
Knowledge of COPD and prehospital oxygen therapy (*n* = 65). (**A**) Proportion of respondents answering each of the seven scored items correctly, with 95% confidence intervals. (**B**) Proportion selecting the originally keyed response for the three items excluded from the knowledge score during revision; these responses are shown for completeness and are not interpreted as correct.

Tobacco smoke was identified as the principal risk factor for COPD by 70.8% of respondents. Among the 65 respondents, the proportion answering each scored knowledge item correctly ranged from 44.6% to 86.2%. The two lowest correct-response rates were observed for infection as the most common cause of COPD exacerbation (44.6%, 95% CI 33.2–56.7) and for a target SpO_2_ range of 88–92% (58.5%, 95% CI 46.3–69.6) (Figure 1). Among the three items excluded from the score, the proportions selecting the originally keyed responses were 56.9% (95% CI 44.8–68.2) for the effect of excessive oxygen on ventilation, 83.1% (95% CI 72.2–90.3) for the item on peak expiratory flow, and 41.5% (95% CI 30.4–53.7) for second-line oxygen-delivery strategy; these are reported for completeness only and were neither included in the knowledge score nor interpreted as correct.

**Figure 2:**
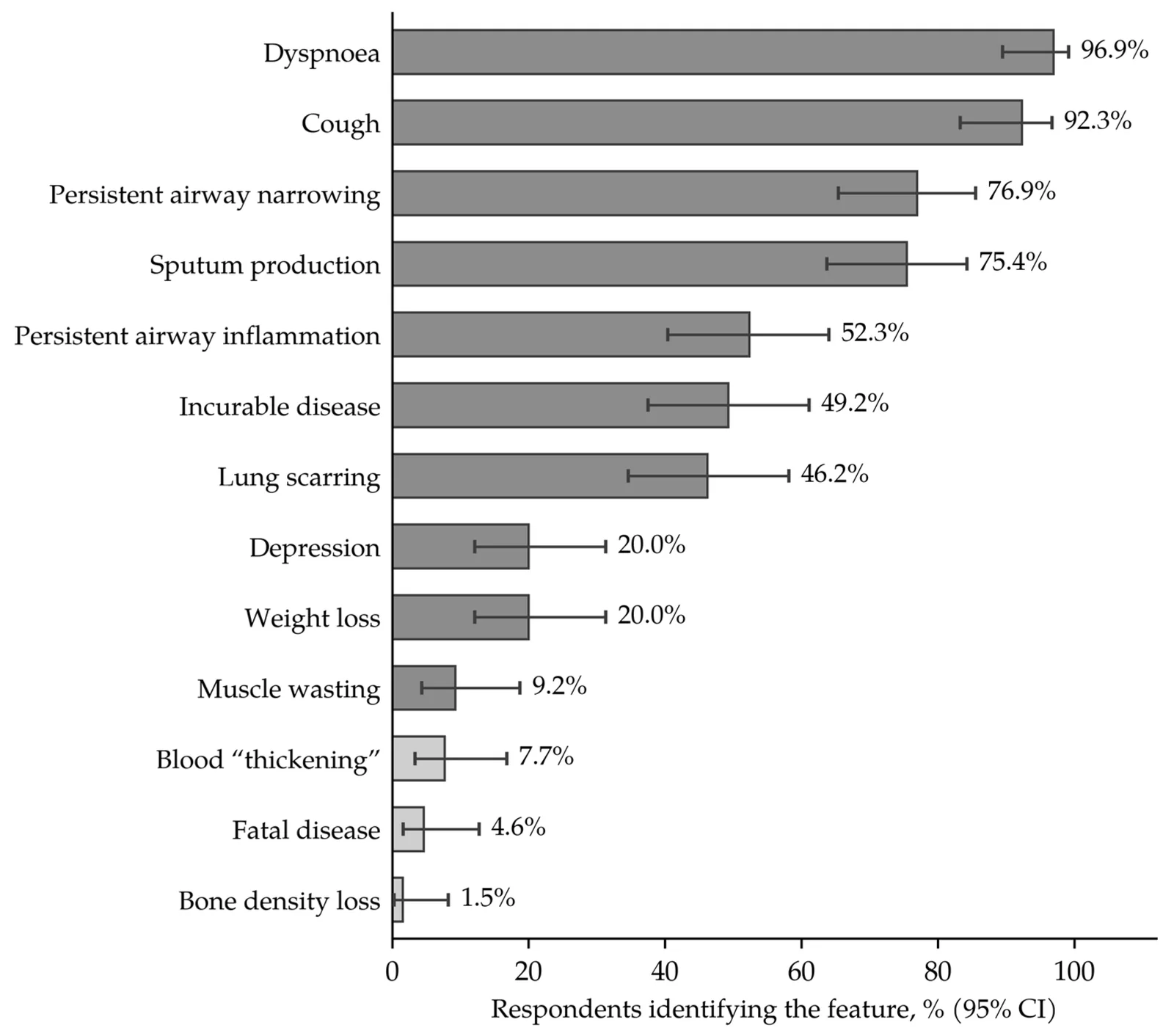
Proportion of respondents who identified each feature as characteristic of COPD (*n* = 65).

When asked which features are characteristic of COPD (multiple answers allowed), respiratory features (dyspnoea, cough, persistent airway narrowing, and sputum production) were identified far more frequently than systemic manifestations such as depression, weight loss, and muscle wasting (Figure 2).

**Figure 3:**
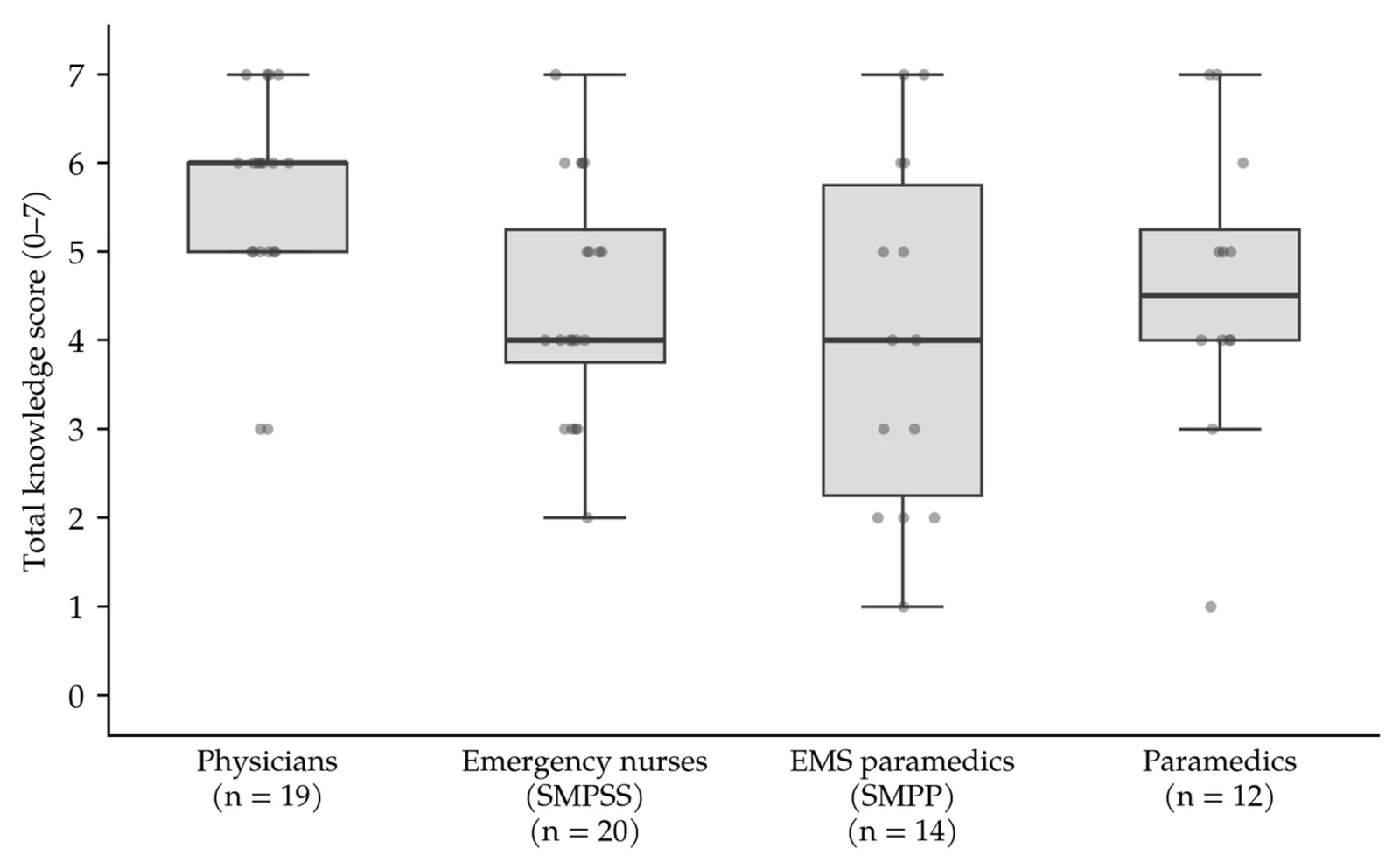
Total knowledge score (0–7) by professional role (*n* = 65). Boxes show the median and interquartile range, whiskers the full range, and points individual respondents. Scores differed across the four professional groups (Kruskal–Wallis test, H = 8.65, df = 3, *p* = 0.034); pairwise comparisons were not performed.

The proportion of correct responses across the scored emergency-management items was 71.9% among physicians, 70.0% among SMPSSs, 69.4% among paramedics, and 61.9% among SMPPs. Knowledge of hypercapnia by professional role ranged from 60.7% among SMPPs to 97.4% among physicians. Overall, respondents answered a median of 5 of the 7 scored knowledge items correctly (IQR, 4–6). Total knowledge scores differed significantly by professional role (Kruskal–Wallis test, *p* = 0.034), with physicians scoring highest (median 6, IQR 5–6) and the other groups lower (median 4–4.5) (Figure 3). Scores did not differ significantly by years of professional experience (*p* = 0.63), sex (*p* = 0.92), or self-reported frequency of contact with COPD exacerbations (*p* = 0.97). Item-level results by professional group are shown in Table 2. In the sensitivity analysis using the original 10-item score, respondents answered a median of 7 items correctly (IQR, 5–8) and the same pattern was observed (professional role *p* = 0.006; experience *p* = 0.70; sex *p* = 0.89; contact frequency *p* = 0.72), indicating that the item exclusions did not alter the findings.

### 3.2. National Trends in Chronic Hypercapnic Respiratory Failure

**Figure 4:**
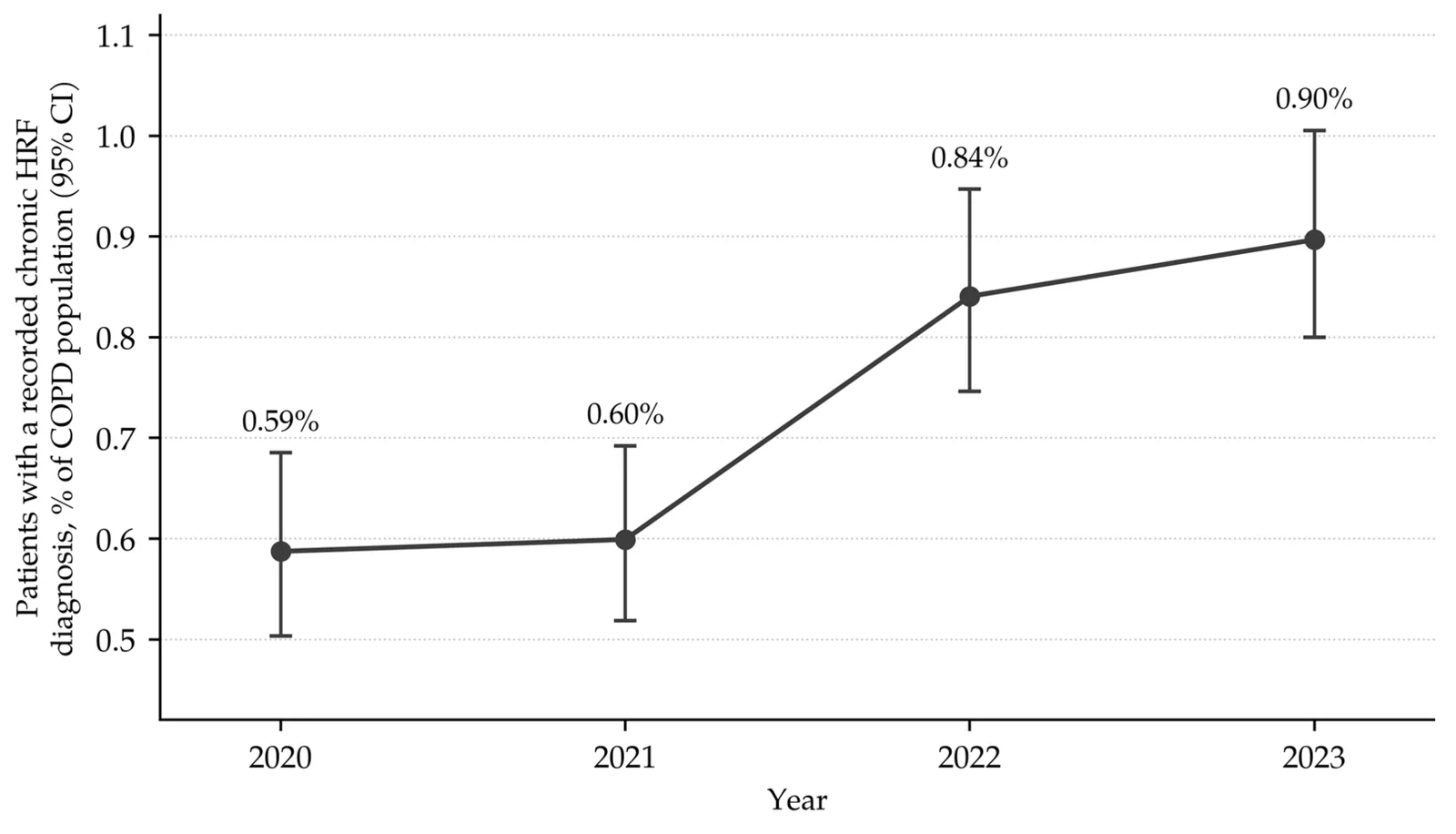
Annual proportion of patients with COPD who had a recorded chronic HRF diagnosis, with 95% confidence intervals, Lithuania, 2020–2023.

Between 2020 and 2023, the recorded COPD population in ESPBI IS increased by 19.1%, from 27,239 to 32,446, and the number of COPD patients with a recorded diagnosis of chronic HRF increased by 81.9%, from 160 to 291 (Table 3). The proportion of COPD patients with a recorded chronic HRF diagnosis rose from 0.59% (95% CI 0.50–0.69) in 2020 to 0.90% (95% CI 0.80–1.01) in 2023, an absolute increase of 0.31 percentage points (95% CI 0.17–0.45), corresponding to a risk ratio of 1.53 (95% CI 1.26–1.85) (Figure 4). Year-to-year differences were statistically significant (χ^2^ = 32.2, df = 3, *p* < 0.001); in Holm-adjusted pairwise post hoc comparisons, all contrasts were significant except for the 2020 versus 2021 and the 2022 versus 2023 comparisons, indicating a step increase between 2021 and 2022 followed by a plateau. The proportion was higher in men than in women in every study year (Table 4). The absolute increase between 2020 and 2023 was larger in women (+0.41 percentage points, 95% CI 0.22–0.60) than in men (+0.24 percentage points, 95% CI 0.05–0.43), so that the difference between the sexes narrowed from 0.39 to 0.22 percentage points, although men remained higher throughout. Pooled across study years, the unadjusted odds of a recorded chronic HRF diagnosis were higher in men than in women (OR 1.71, 95% CI 1.47–1.98, *p* < 0.001). In the age-stratified analysis, the proportion was highest in patients aged 50–59 years (1.17%, 95% CI 1.01–1.35) and fell progressively with increasing age, to 0.37% (95% CI 0.31–0.45) in patients aged 80 years or older (Table 5). In a multivariable model adjusting for age group and sex, male sex remained associated with a recorded chronic HRF diagnosis (adjusted OR 1.55, 95% CI 1.28–1.88, *p* < 0.001), and patients aged 80 years or older had lower adjusted odds than those younger than 50 years (adjusted OR 0.53, 95% CI 0.33–0.85, *p* = 0.008); the remaining age groups did not differ significantly from the reference category.

Across the four annual cross-sections, 902 of 2174 patient-year observations with a recorded J96.11 diagnosis nationally (41.5%) also included a recorded COPD diagnosis in the same calendar year. The annual number of patients with a recorded J96.11 diagnosis increased from 385 in 2020 to 747 in 2023 (+94.0%), and the share accounted for by COPD remained broadly stable, at 41.6% (95% CI 36.7–46.5) in 2020 and 39.0% (95% CI 35.5–42.5) in 2023. Patients with a recorded J96.11 diagnosis and concurrent COPD were older and more often male than those without COPD (Table 6).

## 4. Discussion

The proportion of COPD patients with a recorded diagnosis of chronic HRF increased from 0.59% to 0.90% between 2020 and 2023, representing a substantially greater increase than that observed in the overall COPD population. Previous studies have demonstrated that chronic HRF is common among patients with advanced COPD and is associated with more severe airflow limitation, greater hyperinflation, increased exacerbation burden, and poorer clinical outcomes [3,4,5]. Recent evidence also suggests that chronic hypercapnia develops progressively as lung function deteriorates. Reduced FVC and FEV_1_ have recently been identified as independent predictors of chronic hypercapnia, highlighting that the earlier recognition of patients at risk may facilitate the timely monitoring and initiation of long-term ventilatory support [16]. Several explanations may account for the increase observed in the present study. First, improved recognition of chronic HRF and changes in diagnostic coding practices may have contributed to the increase in recorded diagnoses. In addition, this increase may reflect a growing population of patients with advanced COPD who are at greater risk of developing chronic hypercapnia [4,5]. Accordingly, the observed increase should be interpreted as a trend in recorded diagnoses rather than a confirmed change in true prevalence. The number of patients with a recorded chronic HRF diagnosis increased in both sexes, and the proportion was higher in men than in women in every study year (unadjusted OR 1.71, 95% CI 1.47–1.98; adjusted OR 1.55, 95% CI 1.28–1.88). The relative increase was larger in women, so the difference between the sexes narrowed over the study period, although men remained higher throughout. The proportion of COPD patients with a recorded chronic HRF diagnosis was highest in those aged 50–59 years and fell progressively with age, and the adjusted odds were lowest in patients aged 80 years or older. Whether these patterns reflect genuine differences in disease burden or differences in arterial blood gas testing and diagnostic coding cannot be determined from electronic health record data. To our knowledge, this is the first study to provide national data on chronic HRF among patients with COPD in Lithuania and to examine the knowledge of prehospital emergency-care professionals regarding hypercapnia and oxygen therapy.

Placing the COPD subgroup against all patients with a recorded J96.11 diagnosis nationally provides additional context. Across the four annual cross-sections, COPD accounted for 41.5% of patient-year observations with a recorded J96.11 diagnosis, and this share remained broadly stable between 2020 and 2023. Notably, the number of patients with a recorded J96.11 diagnosis nationally rose by 94.0% over the same period, somewhat more than the 81.9% increase observed within the COPD population, so the trend reported here forms part of a broader national increase in recorded chronic hypercapnic respiratory failure rather than a COPD-specific phenomenon. Patients with a concurrent COPD diagnosis were nevertheless a distinct group: they were older and more often male than patients with a recorded J96.11 diagnosis and no COPD diagnosis (Table 6).

In this study, we assessed knowledge of COPD, hypercapnia, and oxygen therapy among prehospital emergency-care professionals and examined nationwide trends in chronic HRF among patients with COPD in Lithuania. The main findings were threefold. First, knowledge varied across domains, with lower rates of correct responses for several practical aspects of oxygen therapy in patients at risk of hypercapnia. Second, knowledge scores differed across professional groups but were not associated with years of professional experience or self-reported frequency of contact with COPD exacerbations. Third, the proportion of COPD patients with a recorded diagnosis of chronic HRF increased significantly between 2020 and 2023, rising more rapidly than the underlying COPD population.

Appropriate oxygen therapy is a key component of managing patients at risk of hypercapnia, yet deviations from recommended oxygen therapy practices continue to be reported in both prehospital and hospital settings [10,11]. In the present study, practical aspects of oxygen therapy were among the most challenging survey items. Although most respondents correctly identified hypercapnia (78.5%) and its symptoms (72.3%), only 58.5% identified the recommended target oxygen saturation range of 88–92%. Fewer scored items addressed the practical delivery of oxygen after three items were excluded during revision, so this observation rests on a narrower evidential base than in the original analysis and should be interpreted accordingly. Similar knowledge gaps in oxygen therapy have been reported among healthcare professionals in other settings. In a survey from Saudi Arabia, Aloushan et al. identified substantial deficiencies in knowledge and the practice of oxygen therapy among emergency care personnel [12], while Demilew et al. found that only slightly more than half of healthcare professionals demonstrated a good level of knowledge regarding oxygen therapy [13]. Validated questionnaires to assess knowledge of acute oxygen therapy have also been developed, although they are primarily used for hospital-based doctors and nurses rather than prehospital personnel [15]. One possible explanation is that oxygen is often perceived as a relatively safe intervention, and the risks associated with hyperoxia and oxygen-induced hypercapnia may therefore receive less emphasis in routine clinical training and practice.

Beyond oxygen therapy, respondents were more likely to identify classical respiratory manifestations of COPD, such as dyspnoea, cough, sputum production, and persistent airway narrowing, than systemic features, including weight loss, muscle wasting, and depression. These findings are consistent with the contemporary concept of COPD as a heterogeneous multisystem disease rather than an isolated airway disorder. Modern pathogenic models emphasise the interaction between genetic susceptibility, environmental exposures and ageing, resulting in multiple clinical phenotypes and systemic manifestations [17]. Multimorbidity contributes substantially to symptom burden, hospitalisations, and mortality, making the recognition of extrapulmonary manifestations an essential component of COPD management [18]. This finding is noteworthy because COPD is increasingly recognised as a complex multisystem disease rather than a disorder confined solely to the lungs [19]. Patients with COPD frequently experience extrapulmonary manifestations and comorbidities that contribute substantially to symptom burden, quality of life impairment, healthcare utilisation, and prognosis [19,20]. The lower rate of recognition of systemic manifestations observed in our survey may reflect the notion that respiratory symptoms remain the most visible and clinically prominent features of COPD in routine practice, whereas extrapulmonary manifestations and comorbidities receive comparatively less attention during education and clinical training.

Knowledge scores differed across professional groups, with physicians achieving the highest median score, whereas no significant associations were observed with years of professional experience or self-reported frequency of contact with COPD exacerbations. Similar variations in knowledge between professional groups have been reported in previous studies. Aloushan et al. found differences in oxygen therapy knowledge and practice among emergency-care personnel with different professional backgrounds [12]. In a survey of hospital-based physicians in Nigeria, Desalu et al. found that guideline knowledge scores differed significantly by physician grade, with pulmonologists scoring highest and medical officers lowest, although adherence to guideline-based management in clinical practice remained low overall [21]. In contrast, O’Driscoll et al. observed little difference in factual knowledge of emergency oxygen therapy between ambulance crews, nurses, and doctors [14], suggesting that the distribution of knowledge across professional groups may differ between healthcare systems and care settings. However, these findings should be interpreted with caution, as the subgroup analyses in the present study were exploratory and based on relatively small sample sizes. With this caveat, one possible explanation is that formal education and professional training may influence knowledge acquisition more strongly than clinical exposure alone. The pattern was not uniform across items. Physicians answered the hypercapnia items best, correctly identifying the symptoms of hypercapnia in every case, whereas the item on first-line oxygen delivery was answered correctly more often by emergency nurses (95.0%) and EMS paramedics (92.9%) than by physicians (78.9%). This inversion suggests that protocol-driven items may be answered more reliably by the professional groups whose training is organised around them, and that knowledge in this setting is not a single hierarchy.

As the burden of chronic HRF among patients with COPD increases, the ability of prehospital emergency-care professionals to recognise hypercapnia and apply appropriate oxygen therapy may become increasingly important. Patients with acute exacerbations of COPD are frequently assessed and treated by emergency medical services before hospital arrival, and oxygen therapy is among the most commonly administered prehospital interventions [22,23]. Appropriate oxygen titration in this setting may therefore have important implications for subsequent patient management. The two components of this study were conducted in parallel and are reported side by side; the survey respondents are not the clinicians whose patients are represented in the electronic health record data, and the design does not permit any inference linking knowledge levels to the observed temporal trend. Each component is descriptive in its own right, and any relationship between them is hypothesis-generating only. The increasing clinical importance of chronic hypercapnia is further supported by recent population-based evidence showing that achieving adequate control of PaCO_2_ during long-term home mechanical ventilation is independently associated with improved survival rates [24]. These findings reinforce the importance of recognising chronic HRF early in the course of advanced COPD.

The study period overlapped with the COVID-19 pandemic, which may have influenced the observed temporal pattern. Hypercapnia has been reported frequently among invasively ventilated patients with acute hypoxaemic respiratory failure due to COVID-19; however, this evidence relates primarily to acute critical illness and does not establish that SARS-CoV-2 infection causes chronic HRF in patients with COPD [25].

More importantly for the interpretation of the present electronic health record findings, the pandemic substantially altered healthcare utilisation. In Lithuania, avoidable hospital admissions for asthma and COPD declined sharply in 2020 amid restrictions on planned services and an overall reduction in hospital activity, and rose again by 2022 [26]. A large multicentre US study likewise reported a season-matched 53% decline in COPD admissions during the pandemic, associated with a lower burden of community respiratory viral infections [27]. These studies evaluated hospital admissions rather than annual counts of recorded diagnoses, and the two measures need not follow the same temporal pattern.

In the present COPD population, the proportion of patients with a recorded chronic HRF diagnosis was essentially unchanged between 2020 and 2021 (0.59% versus 0.60%; 160 and 183 patients) and increased to 0.84% in 2022. The 2020 versus 2021 and the 2022 versus 2023 contrasts were the pairwise comparisons that did not reach statistical significance, so the temporal pattern was characterised by a step increase between 2021 and 2022 followed by a plateau, rather than by an immediate rise during the first pandemic year. Thus, the available data do not suggest that the overall increase was driven by an immediate pandemic-period rise in recorded HRF among patients with COPD.

Nevertheless, pandemic-related changes in healthcare-seeking behaviour, access to care, respiratory viral circulation, diagnostic activity, case ascertainment, and coding may have influenced the magnitude of the observed trend. Because SARS-CoV-2 infection status and individual-level clinical data were unavailable, these effects could not be quantified.

### Limitations

This study has several limitations that should be considered. First, the electronic health record analysis relied on recorded diagnoses, and the marked increase in recorded chronic HRF may partly reflect changes in diagnostic coding and case ascertainment over time rather than a true rise in incidence. Second, the survey was based on a convenience sample of 65 prehospital professionals from a single country, with the small size of the subgroups (7–8 respondents in the categories with the most experience) limiting statistical power and generalisability. Moreover, because recruitment was conducted through professional social media groups, selection bias cannot be excluded. Third, the questionnaire was developed specifically for this study, and, although based on current guidelines and published literature, it was not formally validated: the knowledge score was an unweighted composite of single-answer items. On review, three items were found to have designated correct answers that could not be unambiguously supported and were excluded from the score; this reduced the number of scored items and, in particular, limited the number of items addressing the practical delivery of oxygen therapy. The absence of formal item validation and pilot testing before administration is a limitation of the survey component. Fourth, the survey group comparisons were exploratory and were not adjusted for multiple testing, so they should be regarded as hypothesis-generating. Fifth, the pooled age- and sex-stratified analyses were based on annual patient counts; patients were unique within each calendar year but may have contributed observations in more than one year, so the pooled figures should be interpreted as patient-year observations rather than as unique individuals over the whole study period. The multivariable model adjusted for age group and sex but not for calendar year. Sixth, respondents were not stratified by medical specialty beyond professional role, and the questionnaire assessed general rather than specialty-specific knowledge; the findings should therefore not be read as a profile of any particular service or specialty. Seventh, the survey reached a convenience sample that cannot be assumed to represent all regions of the country, so the survey findings are not nationally representative, in contrast to the electronic health record component, which covers the whole national population.

## 5. Conclusions

Between 2020 and 2023, the proportion of Lithuanian patients with COPD who had a recorded diagnosis of chronic HRF increased significantly, rising disproportionately relative to the underlying COPD population. In parallel, a survey of prehospital emergency-care professionals revealed variability in knowledge of COPD, hypercapnia, and oxygen therapy, with a lower rate of correct responses for the recommended target oxygen saturation range. Knowledge varied across professional roles but was not associated with years of experience or frequency of contact with COPD exacerbations. The survey and electronic health record components were analysed in parallel and were not designed to assess any relationship between them. The electronic health record findings underscore the need for continued surveillance and recognition of chronic HRF in COPD, whereas the survey findings identify potential areas for further evaluation and targeted education in prehospital oxygen therapy.

## Figures and Tables

**Table 1:** Characteristics of survey respondents (*n* = 65).

Characteristic	*n* (%)
**Professional role**	
Emergency nurses (SMPSS)	20 (30.8)
Physicians	19 (29.2)
EMS paramedics (SMPP)	14 (21.5)
Paramedics	12 (18.5)
**Professional experience**	
5 years or less	33 (50.8)
6–10 years	17 (26.2)
11–20 years	7 (10.8)
21 years or more	8 (12.3)
**Sex**	
Women	46 (70.8)
Men	19 (29.2)
**Frequent contact with COPD exacerbations**	
Overall	43 (66.2)
Physicians	11 (57.9)
Emergency nurses (SMPSS)	13 (65.0)
Paramedics	8 (66.7)
EMS paramedics (SMPP)	11 (78.6)

Percentages for professional role, experience, and sex are of the total sample (*n* = 65); percentages for frequent contact with COPD exacerbations are within each professional group. COPD, chronic obstructive pulmonary disease; EMS, emergency medical services; SMPP, emergency medical care paramedic; SMPSS, emergency medical care nursing specialist.

**Table 2:** Correct responses to scored knowledge items by professional group, *n* (%).

Item	Physicians (*n* = 19)	SMPSS (*n* = 20)	SMPP (*n* = 14)	Paramedics (*n* = 12)
Principal risk factor	16 (84.2)	10 (50.0)	10 (71.4)	10 (83.3)
Target SpO_2_ 88–92%	14 (73.7)	11 (55.0)	6 (42.9)	7 (58.3)
Definition of hypercapnia	18 (94.7)	15 (75.0)	10 (71.4)	8 (66.7)
Symptoms of hypercapnia	19 (100)	13 (65.0)	7 (50.0)	8 (66.7)
First-line device and dose	15 (78.9)	19 (95.0)	13 (92.9)	9 (75.0)
First-line drug	12 (63.2)	12 (60.0)	7 (50.0)	9 (75.0)
Most common cause of exacerbation	12 (63.2)	9 (45.0)	4 (28.6)	4 (33.3)
Total score, median (IQR)	6 (5–6)	4 (3.8–5.3)	4 (2.3–5.8)	4.5 (4–5.3)

Subgroup sizes are small and all between-group comparisons are exploratory and hypothesis-generating. SMPP, emergency medical care paramedic; SMPSS, emergency medical care nursing specialist.

**Table 3:** National electronic health record data on COPD and recorded chronic HRF, Lithuania, 2020–2023.

Year	Patients with COPD, *n*	With Recorded Chronic HRF, *n*	% of COPD Population (95% CI)
2020	27,239	160	0.59 (0.50–0.69)
2021	30,538	183	0.60 (0.52–0.69)
2022	31,881	268	0.84 (0.75–0.95)
2023	32,446	291	0.90 (0.80–1.01)

All counts represent unique patients per calendar year. The proportion of patients with COPD who had a recorded chronic HRF diagnosis increased across the study period (χ^2^ test, *p* < 0.001); in Holm-adjusted pairwise post hoc comparisons, all year contrasts were significant except 2020 vs. 2021 and 2022 vs. 2023. COPD, chronic obstructive pulmonary disease; HRF, hypercapnic respiratory failure.

**Table 4:** Proportion of COPD patients with a recorded chronic HRF diagnosis, by sex and year.

Year	Men, % (95% CI)	Women, % (95% CI)	Difference M−W, pp (95% CI)
2020	0.74 (0.62–0.88)	0.35 (0.25–0.48)	+0.39 (+0.22 to +0.56)
2021	0.75 (0.64–0.89)	0.35 (0.26–0.48)	+0.40 (+0.24 to +0.56)
2022	1.01 (0.88–1.16)	0.57 (0.45–0.72)	+0.44 (+0.25 to +0.63)
2023	0.98 (0.85–1.13)	0.76 (0.62–0.93)	+0.22 (+0.02 to +0.43)

pp, percentage points; CI, confidence interval.

**Table 5:** Recorded chronic HRF among patients with COPD by age group and sex, 2020–2023: proportions and adjusted odds ratios.

Variable	Patients with COPD, *n*	With Chronic HRF, *n*	% (95% CI)	Adjusted OR (95% CI)	*p*
Age < 50 years	5817	44	0.76 (0.56–1.01)	1 (reference)	—
Age 50–59 years	15,178	177	1.17 (1.01–1.35)	1.54 (0.98–2.43)	0.064
Age 60–69 years	35,210	324	0.92 (0.83–1.03)	1.22 (0.79–1.88)	0.38
Age 70–79 years	36,507	247	0.68 (0.60–0.77)	0.92 (0.59–1.42)	0.69
Age ≥ 80 years	29,392	110	0.37 (0.31–0.45)	0.53 (0.33–0.85)	0.008
Women	47,241	244	0.52 (0.45–0.59)	1 (reference)	—
Men	74,863	658	0.88 (0.81–0.95)	1.55 (1.28–1.88)	<0.001

Counts represent patient-year observations pooled across the four annual cross-sections. Odds ratios are mutually adjusted for age group and sex. CI, confidence interval; HRF, hypercapnic respiratory failure; OR, odds ratio.

**Table 6:** Age and sex distribution of patients with a recorded J96.11 diagnosis, by presence of a concurrent COPD diagnosis, 2020–2023.

	With COPD (*n* = 902)	Without COPD (*n* = 1272)
Men, *n* (%)	658 (72.9)	722 (56.8)
Age < 50 years, *n* (%)	44 (4.9)	371 (29.2)
Age 50–59 years, *n* (%)	177 (19.6)	249 (19.6)
Age 60–69 years, *n* (%)	324 (35.9)	403 (31.7)
Age 70–79 years, *n* (%)	247 (27.4)	177 (13.9)
Age ≥ 80 years, *n* (%)	110 (12.2)	72 (5.7)

Counts represent patient-year observations pooled across the four annual cross-sections. The age distribution differed between the groups (χ^2^(4) = 241.9, *p* < 0.001), as did the sex distribution (χ^2^(1) = 59.1, *p* < 0.001; odds ratio for male sex 2.05, 95% CI 1.71–2.47). COPD, chronic obstructive pulmonary disease.

## Data Availability

The survey data analysed in this study are available from the corresponding author on reasonable request. The national health data were obtained from the ESPBI IS national electronic health record system through the Lithuanian State Data Agency under the Law on Health Data Reuse and are subject to that agency’s access and governance regulations; they are not publicly available but may be requested from the agency.

## Abbreviations

The following abbreviations are used in this manuscript:

CI	Confidence interval
CO_2_	Carbon dioxide
COPD	Chronic obstructive pulmonary disease
EMS	Emergency medical services
HRF	Hypercapnic respiratory failure
ICD-10-AM	International Statistical Classification of Diseases and Related Health Problems, 10th Revision, Australian Modification
IQR	Interquartile range
OR	Odds ratio
SMPP	Emergency medical care paramedic
SMPSS	Emergency medical care nursing specialist
SpO_2_	Peripheral oxygen saturation

## Appendix A

The 15-item questionnaire administered to respondents is provided below (translated from Lithuanian). For knowledge items, the correct answer is marked (√); items 1–4 collected demographic and practice information and had no correct answer. Items 7, 8 and 11 were excluded from the knowledge score during revision (see Section 2.1); the answers marked for these items are those used at the time of data collection and are shown for completeness but should not be regarded as validated correct answers.

**Table A1:** Survey questionnaire.

No.	Question	Response Options (√ = Correct Answer)
1	Your role *[demographic]*	Paramedic; Emergency medical care paramedic (SMPP); Emergency medical care nursing specialist (SMPSS); Physician
2	Work experience *[demographic]*	5 years or less; 6–10 years; 11–20 years; 21 years or more
3	Sex *[demographic]*	Female; Male; Other
4	Do you frequently encounter patients with a COPD exacerbation? *[practice; no correct answer]*	Yes; No; Don’t know
5	What is the principal factor promoting the development of COPD?	Genetic predisposition; Bacterial infection; **√ Inhalation of harmful particles, most often tobacco smoke**; Viral infection
6	What is the target oxygen saturation (SpO_2_) range for COPD patients at risk of hypercapnic respiratory failure during oxygen therapy?	95–100%; 92–98%; **√ 88–92%**; 90–94%
7	An excessively dosed oxygen-therapy flow in a COPD patient may…	Increase blood oxygen saturation; **√ Suppress the respiratory centre**; Reduce hypercapnia (ventilate off CO_2_); Increase blood pH
8	Which symptom is NOT common in COPD?	Chronic cough; Sputum production; Dyspnoea; **√ Increased peak expiratory flow**
9	What is hypercapnia?	Lack of oxygen in the blood; **√ Excess carbon dioxide in the blood**; Decreased blood pH; Increased respiratory rate
10	What is the first-choice oxygen-therapy device and dose for a COPD patient in respiratory distress?	**√ Nasal cannula 2–4 L/min**; Reservoir mask 10–15 L/min; Nasal cannula 6–10 L/min; Reservoir mask 2–4 L/min
11	If oxygen is delivered and saturation does not rise, what is the second-choice device and dose?	Nasal cannula 10–15 L/min; Reservoir mask 10–15 L/min; **√ Reservoir mask 6–10 L/min**; Nasal cannula 8–10 L/min
12	In advanced management of a COPD patient in respiratory distress, the first-choice drug is…	Methylprednisolone; **√ Salbutamol**; Dexamethasone; Adrenaline infusion
13	The most common cause of a COPD exacerbation is…	Cigarette smoke; **√ Infection**; Non-adherence to medication; Cold air
14	What are the symptoms of hypercapnia?	**√ Drowsiness/lethargy, cognitive disturbance, headache**; Miosis, polyuria, bradycardia, dry cough; Dry cough, cold sweat, sudden tachyarrhythmia
15	Which of the following are characteristic of COPD? (multiple answers allowed; analysed descriptively—no single correct answer)	Dyspnoea; Cough; Sputum production; Bone density loss; Lung scarring; It is a fatal disease; Weight loss; Blood “thickening”; Persistent airway narrowing; It is an incurable disease; Depression; Muscle wasting; Persistent airway inflammation; Don’t know
